# Supplementary material for: Transdiagnostic Perspective of Impulsivity and Compulsivity in Obesity: From Cognitive Profile to Self-Reported Dimensions in Clinical Samples with and without Diabetes
Source: Nutrients. 2021 Dec 10;13(12):4426. doi: 10.3390/nu13124426 (PMC8707121; doi:10.3390/nu13124426)
Supplement: Supplementary file 1 [file nutrients-13-04426-s001.zip › nutrients-1457662-supplementary.pdf]

Supplementary Materials

Table S1. Comparison of psychopathology state between the groups, according to the SCL-90R scales between the groups: ANCOVA adjusted by sex, age, education and BMI.

	AN-R *N=107*		OB-T2D *N=115*		HC *N=171*		OB+T2D *N=67*		GD *N=121*		Polynomial contrasts Trends *(p-value)*			
	Mean	*SD*	*Mean*	*SD*	*Mean*	*SD*	*Mean*	*SD*	*Mean*	*SD*	*O1*	*O2*	*O3*	*O4*
Somatic	1.28	0.91	1.33	0.82	0.64	0.47	0.99	0.80	1.36	0.81	.567	**.001***	**.004***	**.018***
Obsessive-compulsive	1.24	0.91	1.26	0.73	0.70	0.56	1.04	0.67	1.45	0.87	.505	**.001***	**.016***	.055
Interpersonal sensitivity	1.43	0.97	1.20	0.83	0.57	0.56	0.74	0.60	1.35	0.87	**.049***	**.001***	**.002***	.197
Depressive	1.78	1.00	1.31	0.77	0.57	0.51	0.98	0.76	1.90	0.97	.781	**.001***	**.006***	.107
Anxiety	1.08	0.87	1.04	0.72	0.43	0.40	0.67	0.60	1.35	0.85	.564	**.001***	**.001***	.086
Hostility	0.87	0.84	0.73	0.62	0.38	0.50	0.51	0.46	1.19	0.91	.123	**.001***	**.002***	.604
Phobic anxiety	0.56	0.66	0.45	0.59	0.16	0.26	0.25	0.60	0.58	0.62	.434	**.001***	**.026***	.391
Paranoia	1.09	0.84	0.99	0.69	0.50	0.55	0.72	0.63	1.21	0.82	.921	**.001***	**.008***	.171
Psychotic	1.01	0.72	0.63	0.54	0.28	0.30	0.50	0.85	1.13	0.76	.679	**.001***	.101	.507
GSI	1.24	0.75	1.08	0.63	0.51	0.40	0.77	0.53	1.38	0.73	.917	**.001***	**.001***	.077
PST	49.07	19.77	52.37	19.44	29.23	17.67	42.78	18.45	55.65	20.78	.644	**.001***	**.001***	**.001***
PSDI	2.11	0.61	1.71	0.48	1.46	0.33	1.58	0.52	2.10	0.62	.474	**.001***	.169	.813

**Pairwise comparisons**	AN-R/ OB-T2D	AN-R/ HC	AN-R/ OB+T2D	AN-R/ GD	OB-T2D/ HC	OB-T2D/ OB+T2D	OB-T2D/ GD	HC/ OB+T2D	HC/ GD	OB+T2D/ GD	η²
Somatic	.798	**<.001***	.139	.524	**<.001***	**.004***	.817	**.038***	**<.001***	**.016***	**.134†**
Obsessive-compulsive	.937	**<.001***	.314	.119	**.001***	.073	.218	**.046***	**<.001***	**.010***	**.109†**
Interpersonal sensitivity	.254	**<.001***	**.001***	.579	**<.001***	**<.001***	.356	.324	**<.001***	**<.001***	**.176†**
Depressive	**.021***	**<.001***	**<.001***	.400	**<.001***	**.010***	**<.001***	**.024***	**<.001***	**<.001***	**.289†**
Anxiety	.819	**<.001***	**.027***	**.033***	**<.001***	**.001***	**.036***	.111	**<.001***	**<.001***	**.185†**
Hostility	.432	**<.001***	.054	**.009***	**.021***	.054	**.001***	.430	**<.001***	**<.001***	**.127†**
Phobic anxiety	.427	**<.001***	**.029***	.857	**.009***	**.019***	.261	.443	**<.001***	**.004***	**.101†**
Paranoia	.602	**<.001***	.051	.334	**.001***	**.017***	.144	.174	**<.001***	**.001***	**.124†**
Psychotic	**.016***	**<.001***	**.002***	.304	**.010***	.213	**<.001***	.127	**<.001***	**<.001***	**.196†**
GSI	.294	**<.001***	**.004***	.198	**<.001***	**.002***	**.017***	.058	**<.001***	**<.001***	**.225†**
PST	.501	**<.001***	.218	.055	**<.001***	**.002***	.414	**.002***	**<.001***	**.002***	**.203†**
PSDI	**.002***	**<.001***	**<.001***	.922	**.021***	.115	**<.001***	.298	**<.001***	**<.001***	**.202†**

Note. AN: anorexia nervosa-restrictive. OB-T2D: obese without diabetes. HC: healthy control. OB+T2D: obese with diabetes. GD: gambling disorder. GSI: global severity index. PST: Positive Symptom Total. PSDI: Positive Symptom Distress Index. SD: standard deviation. O1: Order 1, linear. O2: order 2, quadratic. O3: order 3, cubic. O4: order 4, quartic. η^2: Partial eta-squared. *Bold: significant parameter. †Bold: effect size within the ranges moderate-medium to large-high.

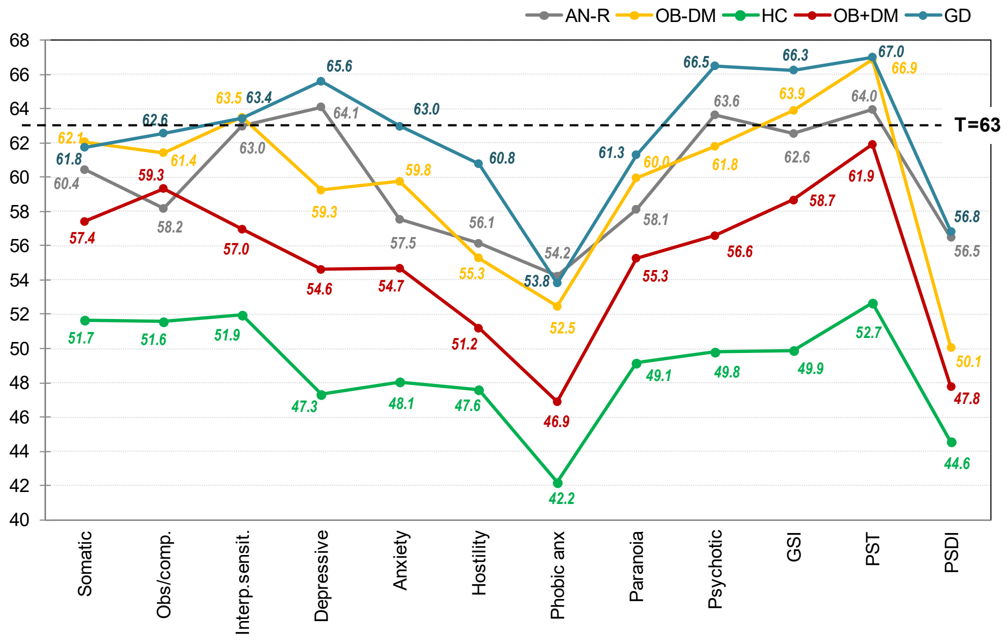

Figure S1. Line-charts with the SCL-90R profile in the study (mean T-scores). Note. AN-R: anorexia nervosa restrictive. OB-T2D: obese without diabetes. HC: healthy control. OB+T2D: obese with diabetes. GD: gambling disorder. GSI: global severity index. PST: Positive Symptom Total. PSDI: Positive Symptom Distress Index. Y-axis represents the mean for the T-scores (adjusted by sex, age, education, and BMI).
